# Automated delineation of putative non-contrast-enhancing tumor in glioblastoma: Prognostic insights

**DOI:** 10.1093/neuonc/noag042

**Published:** 2026-02-25

**Authors:** Maria Gómez-Mahiques, Carles Lopez-Mateu, F Javier Gil-Terrón, Victor Montosa-I-Micó, Siri Fløgstad Svensson, Eduardo Erasmo Mendoza Mireles, Einar Osland Vik-Mo, Kyrre E Emblem, Carme Balañà-Quintero, Josep Puig, Cristina Alenda, Elena Martinez-Saez, Fran Martínez-Ricarte, Marta Quirós-Martí, Vicent Quilis-Quesada, Juan M García-Gómez, Elies Fuster-Garcia

**Affiliations:** Instituto Universitario de Tecnologías de la Información y Comunicaciones (ITACA), Universitat Politècnica de València, Valencia, Spain; Instituto Universitario de Tecnologías de la Información y Comunicaciones (ITACA), Universitat Politècnica de València, Valencia, Spain; Instituto Universitario de Tecnologías de la Información y Comunicaciones (ITACA), Universitat Politècnica de València, Valencia, Spain; Instituto Universitario de Tecnologías de la Información y Comunicaciones (ITACA), Universitat Politècnica de València, Valencia, Spain; Department for Physics and Computational Radiology, Division of Radiology and Nuclear Medicine, Oslo University Hospital, Oslo, Norway; Vilhelm Magnus Laboratory, Department of Neurosurgery, Oslo University Hospital, Oslo, Norway; Institute of Clinical Medicine, Faculty of Medicine, University of Oslo, Oslo, Norway; Vilhelm Magnus Laboratory, Department of Neurosurgery, Oslo University Hospital, Oslo, Norway; Institute of Clinical Medicine, Faculty of Medicine, University of Oslo, Oslo, Norway; Department for Physics and Computational Radiology, Division of Radiology and Nuclear Medicine, Oslo University Hospital, Oslo, Norway; Institut d’Investigació Germans Trias i Pujol, Barcelona, Spain; Department of Radiology and IDIBAPS, Hospital Clínic de Barcelona, Barcelona, Spain; Pathology Department, Alicante Department of Health—General Hospital (C.A.); Alicante Institute for Health and Biomedical Research (ISABIAL), Alicante, Spain; Servicio de Anatomía Patológica, Hospital Universitari Vall d’Hebron, Barcelona, Spain; Servicio de Neurocirugía, Hospital Universitari Vall d’Hebron, Barcelona, Spain; Servicio de Neurocirugía, Hospital Clínic Universitari de València, València, Spain; Servicio de Neurocirugía, Hospital Clínic Universitari de València, València, Spain; Departamento de Cirugía, Facultat de Medicina, Universitat de València, València, Spain; Department of Neurosurgery, Mayo Clinic, Jacksonville (V.Q-Q.); Instituto Universitario de Tecnologías de la Información y Comunicaciones (ITACA), Universitat Politècnica de València, Valencia, Spain; Instituto Universitario de Tecnologías de la Información y Comunicaciones (ITACA), Universitat Politècnica de València, Valencia, Spain

**Keywords:** glioblastoma, non-contrast-enhancing tumor, overall survival, supramaximal resection, T2/FLAIR heterogeneity

## Abstract

**Background:**

Precise delineation of non-contrast-enhancing tumor (nCET) in glioblastoma (GB) is critical for maximal safe resection, yet routine imaging cannot reliably separate infiltrative tumor from vasogenic edema. The aim of this study was to develop and validate an automated method to identify peritumoral subregions compatible with nCET and assess its prognostic value.

**Methods:**

Pre-operative T2-weighted and FLAIR MRI from 940 patients with newly diagnosed GB in four multicenter cohorts were analyzed. A deep-learning model segmented enhancing tumor, edema and necrosis; a non-local, spatially varying finite mixture model was applied to identify edema subregions characterized by relatively lower FLAIR hyperintensity, hypothesized to reflect nCET-related tissue. The ratio of these subregions to total edema volume defined the T2/FLAIR Heterogeneity Index (TFHI). Associations between TFHI and overall survival (OS) were examined with Kaplan–Meier curves and multivariable Cox regression.

**Results:**

Higher TFHI values stratified patients with shorter OS. In the NCT03439332, TFHI above the optimal threshold was associated with a twofold increased hazard of death (hazard ratio [HR] 2.07, 95 % confidence interval 1.33-3.21; *P *= .0013) and a reduction in median survival of 98 days. Significant, though smaller, prognostic effects were confirmed in GLIOCAT & BraTS (HR= 1.37; *P* = .047), OUS (HR = 1.37; *P* = .0032) and pooled analysis (HR= 1.26; *P* = .0008). TFHI remained an independent predictor after adjustment for age, extent of resection and *MGMT* methylation.

**Conclusions:**

We present a reproducible, server-hosted tool for automated identification of imaging-defined, putative nCET-related peritumoral subregions and TFHI biomarker extraction that enables independent prognostic stratification. This approach provides a quantitative framework for studying peritumoral heterogeneity in GB.

## Graphical Abstract

**Graphical Abstract:**
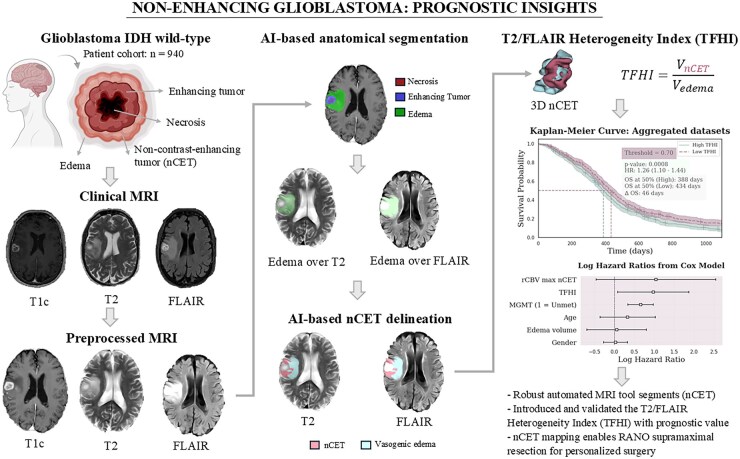

Key Points

- KP1: Robust MRI tool identifies putative non-contrast-enhancing (nCET) regions in glioblastoma.
- KP2: Introduced and validated the T2/FLAIR Heterogeneity Index (TFHI) with prognostic value.
- KP3: nCET mapping aligns with RANO supramaximal resection for personalized surgery.

Importance of the Study

This study underscores the clinical importance of accurately identifying non-contrast-enhancing tumor (nCET) regions in glioblastoma using standard MRI. Despite their lack of contrast enhancement, nCET areas often harbor infiltrative tumor cells critical for disease progression and recurrence. By integrating deep learning segmentation with a non-local finite mixture model, we developed a reproducible, automated methodology for identification of putative nCET and introduced the T2/FLAIR Heterogeneity Index (TFHI), a novel imaging biomarker. Higher TFHI values were associated with reduced survival across cohorts. These findings highlight the prognostic relevance of imaging-defined infiltration patterns and support the use of putative nCET segmentation in clinical decision-making. Importantly, this methodology is conceptually aligned with recent RANO criteria on supramaximal resection and offers a quantitative framework to support research investigating peritumoral heterogeneity in relation to extent of resection. In doing so, our work advances efforts toward more personalized neuro-oncological care, potentially improving outcomes while minimizing functional compromise.

Gliomas are among the most prevalent types of primary brain tumors, accounting for over 70% of all malignant brain tumors.^1^ Glioblastoma (GB) is a particularly aggressive tumor of the central nervous system, with a median survival of only 12-14 months following diagnosis,^1^ despite standard of care that includes tumor resection followed by radiotherapy and concomitant and adjuvant cycles of temozolomide.^2^

In the quest to improve the prognosis for patients with GB, different extents of surgical resection have been explored. Studies have demonstrated that supramaximal resection of a MRI-based abnormality zone beyond the contrast-enhancing tumor (CET) is associated with prolonged patient survival.^3^^,^^4^ Consequently, while this practice is currently recommended as a guideline,^5^^,^^6^ it does not explicitly define which regions should be included in the supramaximal resection, leaving it to the discretion of the neurosurgeon.^5^ Some experts suggest resecting 1-2 cm of tissue around the CET, thus targeting a part of the surrounding edematous region, which is morphologically discernible on conventional MRIs.^5^ This region is biologically heterogeneous, and the surgical approach inevitably involves removing both malignant and non-pathological tissue, which may be detrimental to the patient’s quality of life and could potentially impact their clinical outcome.

In this context, the non-contrast-enhancing tumor (nCET) region, extending beyond the contrast-enhanced areas, plays a crucial role. While challenging to delineate, it can often be identified using T2-weighted fluid-attenuated inversion recovery (FLAIR) and T2-weighted (T2), MRI.^7^^,^^8^ nCET has been described to have a cell density comparable to that of CET.^9^ Incomplete resection of the nCET region has been linked to earlier tumor recurrence.^2^ In contrast, supramaximal resection, which includes the removal of both CET and part of the surrounding FLAIR hyperintensity, has been associated with improved survival. Pessina et al^10^ reported a median survival of 29 months for patients undergoing supramaximal resection, compared to 16 months for those with only macroscopic total resection (>90% of CET). However, a standardized, reproducible approach for defining and segmenting the nCET region has yet to be established, posing a challenge for consistent diagnosis and treatment planning.^8^ Recent reviews by the Response Assessment in Neuro-Oncology (RANO) resect group^6^ and Annette et al^11^ further reinforced these findings, analyzing multiple studies on the impact of resection extent in GB. The authors concluded that achieving a supramaximal resection—defined as the complete removal of CET with ≤ 5 cm³ of residual nCET^6^—correlates with a better prognosis. However, they highlight the complexity of quantifying resection extent, particularly in distinguishing tumor-infiltrated tissue from vasogenic edema on T2 and FLAIR MRIs. RANO’s study underscores the absence of a standardized method to delineate nCET boundaries, stressing the risks of indiscriminately resecting vasogenic edema regions, potentially impacting the patient’s functional capacity without patient benefit.

Our study aimed to develop a precise and reproducible methodology for identifying the nCET region on T2 and FLAIR MRIs, adopting one of the established approaches described in the literature that defines nCET based on spatial variations in signal intensity, particularly relative differences in T2/FLAIR hyperintensity.^7^ By associating the nCET definition outcomes with the overall survival (OS) of GB patients, we sought to underline the clinical relevance of accurately identifying this region. Our approach is designed to serve as a practical tool, particularly for planning supramaximal resections, ultimately providing neurosurgeons with a more informed basis for surgical planning and striving to improve prognostic outcomes for patients with GB.

## Methods

### Patient Cohorts

The following section presents a comprehensive overview of the datasets employed in our study. Each dataset was derived from multiple clinical institutions operating under ethically approved protocols.

#### NCT03439332 Dataset

This study included data from GB patients from seven European clinical centres: Hospital Universitario de La Ribera (Alzira, Spain), Hospital de Manises (Manises, Spain), Hospital Clínic (Barcelona, Spain), Hospital Universitario Vall d’Hebron (Barcelona, Spain), Azienda Ospedaliero-Universitaria di Parma (Parma, Italy), Centre Hospitalier Universitaire de Liège (Liège, Belgium), and Oslo University Hospital—OUS (Oslo, Norway).^12^ Patients were diagnosed with GB grade IV 2016 WHO with histopathological confirmation and followed Stupp standard treatment. The dataset includes comprehensive clinical, molecular, and MRI data: T1-weighted (T1), contrast-enhanced T1-weighted (T1c), T2-weighted (T2), Fluid Attenuated Inversion Recovery (FLAIR), and Dynamic Susceptibility Contrast perfusion (DSC). After applying the eligibility criteria (see below), 113 patients were retained for analysis.

#### GLIOCAT Dataset

This dataset consists of patients from six different institutions: Instituto Catalán de Oncología (ICO) de Badalona (Barcelona), Hospital del Mar (Barcelona), Hospital Clínic (Barcelona), ICO Hospitalet (Barcelona), ICO Girona (Girona), and Hospital Sant Pau (Barcelona). The cohort is part of the retrospective multicentric Gliocat study,^13^ derived from the Gliocat Project, and includes patients diagnosed with GB according to the 2021 WHO CNS tumor classification. All patients received standard first-line treatment, consisting of surgery followed by radiotherapy with concurrent and adjuvant temozolomide, between 2004 and 2015. Additionally, the dataset includes comprehensive clinical, molecular, and MRI (T1, T1c, T2, FLAIR, and DSC) data. A total of 118 patients met the inclusion criteria.

#### BraTS Dataset

A publicly available dataset from the Multimodal Brain Tumor Segmentation Challenge 2019 (BraTS 2019), organized as part of the international MICCAI 2019 conference.^14–16^ This dataset comprises multi-parametric MRI (T1, T1c, T2, and FLAIR) scans and limited clinical data from patients diagnosed with GB. Specifically included in the training corpus for the BraTS 2019 OS prediction challenge. It integrates data from the 2013 BraTS dataset and additional scans from the Center for Biomedical Image Computing and Analytics (CBICA) and The Cancer Imaging Archive (TCIA). This yielded 211 eligible patients.

#### OUS Dataset

The dataset has been used in a previous publication^17^ and based upon a retrospective cohort of glioblastoma patients at OUS). Since 2003, all patients undergoing first-time surgery have been prospectively registered. For our study we included all patients who (1) were diagnosed with a histopathologically verified supratentorial GB (2003-2016), GB WHO grade IV (2016-2019), or tumors classified as gliosarcoma, giant cell GB, or epithelioid GB, according to the relevant WHO classification of tumors of the central nervous system at the time; (2) had undergone surgical resection of the tumor between 2003 and 2020 and (3) had a preoperative anatomical MRI (T1, T2, and FLAIR) scan. The final cohort comprised 498 eligible patients.

#### Criteria Selection

The selection criteria for patient inclusion in our retrospective study were:

1. Adults (≥18 years) with a histopathological diagnosis of GB were included. While aiming to align with the WHO CNS5 (2021) classification, the retrospective nature of the cohort—collected prior to routine molecular profiling—limited the availability of molecular data. To ensure consistency, cases with confirmed IDH1 mutation were excluded (∼2% of the cohort). For patients lacking molecular information, inclusion was determined based on radiological criteria characteristic of IDH1-wildtype GB,^18^ following expert imaging review by a senior investigator with extensive experience in glioma imaging. As part of this review, volumetric relationships between enhancing tumor and peritumoral edema were assessed to identify outlier imaging patterns atypical for IDH1-wildtype glioblastoma. Cases exhibiting disproportionately large edema relative to enhancing tumor volume were flagged for further inspection and were conservatively excluded (*n* = 23) when overall imaging features raised concern for a potential IDH-mutant phenotype. Given the low prevalence of *IDH1* mutations (∼5%-10%) and the predominance of wildtype cases in pre-2021 cohorts, this approach was deemed appropriate to approximate the current WHO definition.^19^^,^^20^
2. A minimum survival period of 30 days post-surgery.
3. Total, supra-total or sub-total surgical resection, excluding biopsies or non-operable cases.
4. Availability of essential MRI studies obtained by 1.5-T or 3-T scanners, depending on the specific purpose of each section. This includes morphological images: T1, T1c, T2, FLAIR images, and, when available, perfusion information from DSC MRI.

### Glioblastoma Segmentation

Initially, the delineation of GB lesions was performed using the anatomical MRIs, categorizing them into three distinct tissues: (1) CET, (2) edema, and (3) necrosis. This procedure includes preliminary preprocessing of the MRI scans, subsequently followed by segmentation employing convolutional neural networks (CNNs):

#### MRI Preprocessing

MRI preprocessing involved several automated steps: all sequences were resampled to 1 mm³ isotropic voxels via linear interpolation, denoised using adaptive non-local means filtering,^21^ and rigidly registered within each patient to the contrast-enhanced T1-weighted image using ANTs.^22^ Subsequently, images were affine-transformed to MNI space^23^ (ANTs), brain-extracted via a U-Net CNN pipeline and corrected for magnetic field inhomogeneity with N4^24^ bias-field correction.

#### Lesion Segmentation

A U‑Net CNN model ^25^ processes 3D patches (32³ voxels) from T1c, T2, and FLAIR images across five hierarchical levels. Each level features four basic blocks (conv–batch norm–ReLU) followed by four residual blocks (adding a conv–batch norm–ReLU with skip connections). Filters increase from 16 to 256 with 3 × 3 × 3 kernels, incorporating down‑ and upsampling for multi‑scale feature capture. Trained on BraTS,^15^ it achieved median Dice scores of 0.85 for contrast‑enhancing tumor and 0.92 for total tumor, using L2 regularization and balanced batches to reduce overfitting and class imbalance.^25^

### nCET Identification

The subsequent step involved segmenting the edema into two subregions: (1) a low-FLAIR/high-T2 subregion hypothesized to correspond to nCET and (2) vasogenic edema. This segmentation was based on the intensity variations observed on T2 and FLAIR MRIs, following the principles outlined by Lasocki et al.^8^ Specifically, the putative nCET-related subregion was operationally defined as having relatively mild intensity on FLAIR and hyperintensity on T2, which may reflect its higher cellular density, as previous studies have shown an inverse correlation between cell density and FLAIR signal intensity.^26^ In contrast, vasogenic edema is hyperintense on both sequences.^7^^,^^8^

Preprocessed T2 and FLAIR MRIs were used as inputs. Within the FLAIR-abnormality mask obtained in the preceding morphological step (where the hyperintense region—including putative nCET-related tissue—is labeled as edema), the low-FLAIR/high-T2 subregion hypothesized to correspond to nCET was segmented using an unsupervised Non-Local Spatially Varying Finite Mixture Model^27^ (NLSVFMM). This algorithm combines SVFMMs—which model local intensity distributions with spatially varying mixture priors—and Non-Local Means (NLM)—a patch-based regularization that promotes similar labels for voxels with similar appearance—to separate imaging-defined edema subregions while accounting for anisotropic local features. As an unsupervised, label-free approach, it does not rely on expert-annotated ground truth or train/test splits; instead, it identifies intrinsic T2/FLAIR heterogeneity within the FLAIR-abnormality mask.

Following NLSVFMM segmentation, we applied a single targeted post-processing constraint to correct partial-volume artifacts arising from resolution differences between T2 and FLAIR. Specifically, within a narrow 4-mm band measured from the outer FLAIR boundary toward the edema, small isolated nCET islands, defined as 26-connected clusters <100 voxels, were removed only when this edge band was located >10 mm from the contrast-enhancing tumor (CET).^8^ To avoid altering morphology in small lesions, this boundary-cleaning step was applied only in cases with large tumors when the inspected 4-mm edge band was located >10 mm from the CET.

Finally, to statistically confirm differences in intensity values between the identified putative nCET region and the remaining vasogenic edema, a Mann-Whitney *U* test was applied, considering results with *P* < .01 as significant. Additionally, to ensure that these differences were practically relevant, we introduced a secondary criterion based on the relative intensity difference. Specifically, we employed Equation (1), where $I_{\mathrm{nCET}}\;$represents the intensity values within the nCET region, and $I_{\mathrm{Vasog}.\;\mathrm{Edema}}$ represents the intensity values within the vasogenic edema region. This metric calculates the percentage difference between nCET and vasogenic edema intensities, normalized by the overall intensity range of the entire brain:


(1) $$\%\Delta I=\frac{\left|I_{\mathrm{nCET}}-I_{\mathrm{Vasog}.\;\mathrm{Edema}}\right|}{\max\left(I_{\mathrm{Brain}}\right)-\min\left(I_{\mathrm{Brain}}\right)}\cdot 100$$

To be deemed practically significant, %ΔI must exceed 5%, a cutoff chosen to balance the subtle T2/FLAIR differences reported for putative nCET vs. vasogenic edema on conventional MRI^8^ with the need to suppress small, noise-driven variations.

### Definition of the T2/FLAIR Heterogeneity Index (TFHI)

To quantify the relevance of the imaging-defined, putative nCET region for specific patients, we defined a novel biomarker derived from its volumetric properties, named the T2/FLAIR Heterogeneity Index (TFHI). This reflects the proportion of the total peritumoral edema volume occupied by putative nCET, highlighting T2/FLAIR heterogeneity consistent with possible infiltration beyond the enhancing core, while not constituting cellular-level proof.

To compute the TFHI, we obtained the volumetric measurements of the imaging-defined nCET and the total edema. The TFHI was then defined as the ratio of the nCET volume ($V_{\mathrm{nCET}}$) to the total edema volume ($V_{\mathrm{edema}}$), as shown in Equation (2).


(2) $$\mathrm{TFHI}=\frac{V_{\mathrm{nCET}}}{V_{\mathrm{edema}}}\;$$

**Figure 1.**
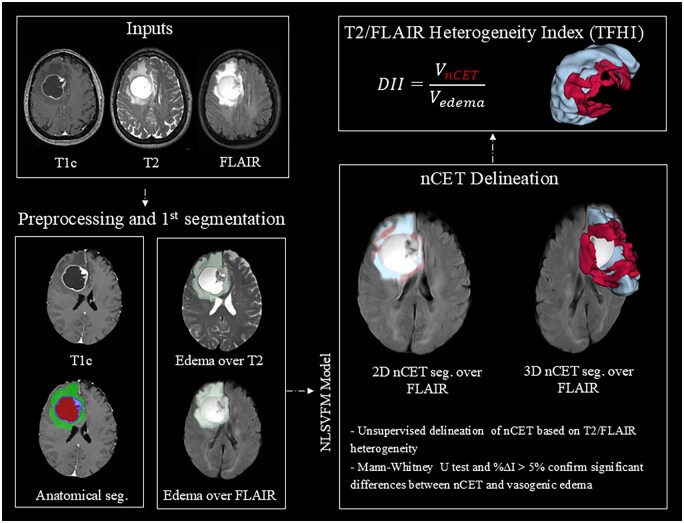
Graphical representation of the pipeline implemented for putative nCET identification and TFHI extraction. First, the input MR images are processed, and the anatomical segmentation is obtained using a U-NET-based approach^25^ within the ONCOhabitats pipeline. This segmentation differentiates necrosis (red), CET (purple), and edema (green). Next, the NLSVFM model is applied to distinguish between vasogenic edema (blue) and voxels exhibiting relatively lower FLAIR signal and higher T2 intensity (red), hypothesized to correspond to nCET, within the segmented edema region, incorporating statistical and practical validations through predefined constraints. Finally, the TFHI was computed, quantitatively characterizing tumor infiltration and invasive heterogeneity.

Figure 1 illustrates the proposed pipeline for imaging-based nCET identification and TFHI extraction, including the preprocessing of MRI scans, anatomical segmentation using the ONCOhabitats U-Net-based approach, edema subsegmentation with the NLSVFM model, and final computation of the TFHI.

### Assessment of TFHI Prognostic Capabilities

We assessed the prognostic capacity of the TFHI biomarker by evaluating its association with OS through univariate and multivariate cox regression analyses.

#### Univariate Analysis

The association between the TFHI and OS was analyzed using univariate Cox regression, aiming to determine an optimal threshold that maximizes the concordance index (c-index) for stratifying patients into high- and low-survival groups. Subsequently, Kaplan–Meier curves were introduced to visually assess differences in survival probability distributions between the groups. The hazard ratio and *P*-value (Wald test) were also computed to quantify these differences.

In parallel, the same analytical approach was applied to the ratio between the volume of the CET and edema (CET/Edema), noting that CET volume is a widely used imaging-derived biomarker with well-established prognostic value.^11^^,^^28^^,^^29^ Whereas CET/Edema captures the bulk of the enhancing tumor core—tissue that is typically removed during surgery—the TFHI quantifies T2/FLAIR heterogeneity within the non-enhancing abnormality, which often persists after resection and is consistent with possible infiltration, though not a cellular-level proof. Evaluating both biomarkers with identical methodology therefore allows a direct, like‑for‑like comparison between a conventional surgical target and our newly proposed T2/FLAIR heterogeneity target, clarifying the additional prognostic information that TFHI may offer for pre‑operative risk stratification and extent‑of‑resection planning. To contextualize overlap between the ratios, we also computed Pearson correlations between CET/edema and TFHI.

Both the TFHI and the CET/Edema volume ratio were evaluated using data from the multi-center studies NCT03439332, GLIOCAT & BraTS, and OUS. These analyses were performed both independently for each dataset, and replicated using a combined, aggregated dataset.

#### Multivariate Analysis

A multivariate Cox regression (with *P*-values derived from Wald tests) was conducted to determine whether the prognostic influence of the TFHI on OS remained independent of routinely employed clinical variables. This analysis incorporated available clinical information, as well as volumetric and perfusion variables from MRI data part of the multicentric study NCT03439332 and GLIOCAT. The BraTS and OUS data were not included in this analysis because of missing clinical and perfusion data that were not amenable to imputation.

Clinical variables (gender, age, *MGMT* status) and imaging features (edema volume, 90th-percentile relative cerebral blood volume (rCBV) in nCET, TFHI) were encoded numerically—*MGMT* methylated = 0, unmethylated = 1, missing imputed by prevalence—and continuous variables scaled to [0,1]. Multivariate Cox models were built incrementally (clinical → +volumetric → +functional) with and without TFHI to assess its added prognostic value. Additionally, univariate Cox analyses of nCET and edema volumes were conducted on each of these factors individually.

### Histopathological Sampling of MRI-Defined nCET

To complement MRI-based analyses with biological context, tissue specimens were obtained from MRI-defined nCET regions in adult patients with pathologically confirmed GB within the framework of the SINUE project (ClinicalTrials.gov NCT07111195). Preoperative imaging-defined nCET masks were exported and loaded into the surgical navigation platforms (BRAINLAB Elements and Medtronic). Biopsy trajectories and targets were planned accordingly. To minimize intraoperative brain shift, sampling was performed before tumor resection. In cases guided with BRAINLAB, the planning MRI was fused with intraoperative ultrasound for navigation; in Medtronic-guided cases targeting relied on preoperative MRI. Specimens were processed with standard hematoxylin and eosin (H&E) staining. Two neuropathologists independently reviewed the slides (blinded to TFHI values) and documented features consistent with infiltrating glioblastoma when present.

### Software

The MRI preprocessing and anatomical segmentation were performed using the libraries presented by Juan-Albarracín et al.^25^^,^^30^ The preprocessing pipeline and the nCET identification approach are fully integrated and accessible through the nCET identification service available at https://www.oncohabitats.upv.es/non-contrast-enhancing-tumor-segmentation/. Finally, the statistical analyses were implemented in Python (v3.8).

## Results

### Study Cohort

**Figure 2.**
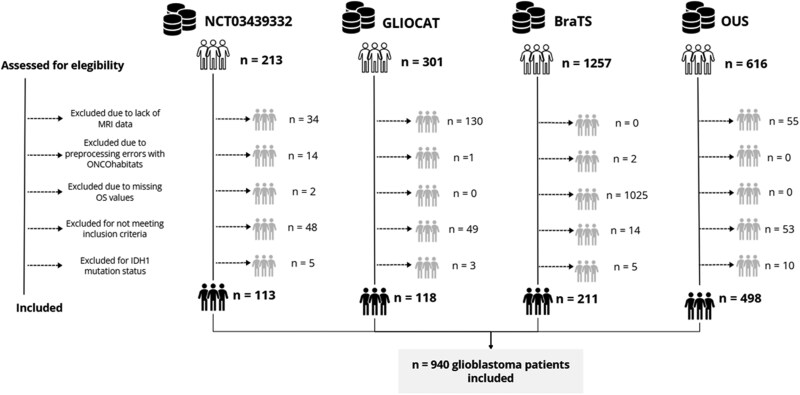
CONSORT diagrams for the NCT03439332, GLIOCAT, BraTS, and OUS datasets. Excluded patients with IDH1 mutations include those identified through molecular analysis, as well as those excluded based on radiological features not consistent with the IDH1-wildtype GB phenotype.

Figure 2 presents the CONSORT diagram illustrating the patient selection process based on the predefined inclusion criteria, detailing the stepwise approach applied to construct the final study cohort and the challenges encountered when applying the defined pipeline.

The final study cohort consisted of 940 patients diagnosed with GB, sourced from four datasets: 113 from NCT03439332, 118 from GLIOCAT, 211 from BraTS, and 498 from OUS. The median age (range) in years was 60 [35-81] for NCT03439332, 60 [32-80] for GLIOCAT, 62 [28-87] for BraTS, and 62 [28-87] for OUS. Median OS (range) in days was 406 [43-1229], 484 [70-2148], 386 [50-1767], and 411 [42-4919], respectively. Regarding survival status, 85 patients from NCT03439332 and 98 from GLIOCAT were deceased at the time of data collection, while survival data were not available for 1025 patients from BraTS. In the OUS cohort, 482 patients had died and 26 were censored.

### nCET Identification

**Figure 3.**
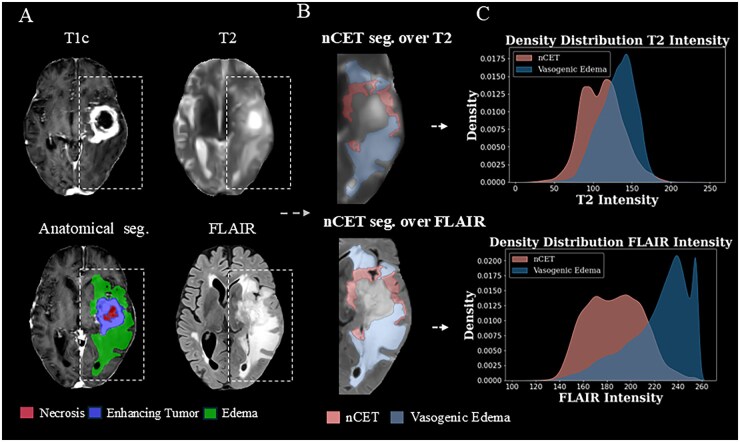
(A) Results of nCET identification obtained from T2 and FLAIR MRIs, displayed alongside the anatomical tumor segmentation. (B) Visualization of the putative nCET and vasogenic edema regions overlaid on T2 and FLAIR sequences. (C) Distribution of signal intensities in T2 and FLAIR for each region—example shown for patient OP03 from the NCT03439332 cohort.

The segmentation of edema into two distinct imaging-defined regions yielded voxel clusters hypothesized to correspond to putative nCET-related tissue and vasogenic edema. As depicted in Figure 3A and B, the LMSVFM algorithm was primed at distinguishing areas of mild image intensity in FLAIR and hyperintensity in T2, setting them apart from regions where both T2 and FLAIR sequences exhibited hyperintense values.

Furthermore, the density distribution curves of intensity values in T2 and FLAIR in Figure 3C reveal a distinct pattern between the two regions. In the FLAIR sequence, the distributions corresponding to the putative nCET-related subregion and vasogenic edema exhibit notable heterogeneity, facilitating their separation. In contrast, the intensity distributions in T2 appear more homogeneous across both regions, leading to a high degree of overlap. This highlights the critical role of the FLAIR sequence in distinguishing nCET from vasogenic edema.

Mann-Whitney U tests for assessing the unsupervised segmentation revealed significant differences between the voxel distributions of the putative nCET and vasogenic edema across the study’s patient cohort in the three datasets: NCT03439332 (*N* = 113), Gliocat (*N* = 118), BraTS (*N* = 211), and BrainPower (*N* = 498) with a total *N* = 940 (*P*-value< .01). The practical implications of this segmentation were evaluated by the %ΔI, as defined in *Equation* (1), revealing that for 917 out of 940 patients (97.5%)—102 from the NCT03439332, 109 from Gliocat, 208 from BraTS and 498 from BrainPower—the segmentation achieved an intensity variation between the putative nCET and the vasogenic edema greater than 5%.

In the correlation analysis between the TFHI and the volumetric ratio of the CET to edema (CET/Edema), we observed that these two imaging-derived features are moderately correlated, with a Pearson correlation coefficient of *r* = 0.51. These results underscore that the CET, located within the tumor core and typically resected during surgery, is quantitatively associated with a peripheral region embedded in the edema—the nCET—automatically delineated through unsupervised segmentation.

### Assessment of TFHI Prognostic Capabilities

#### Univariate Analysis

**Figure 4.**
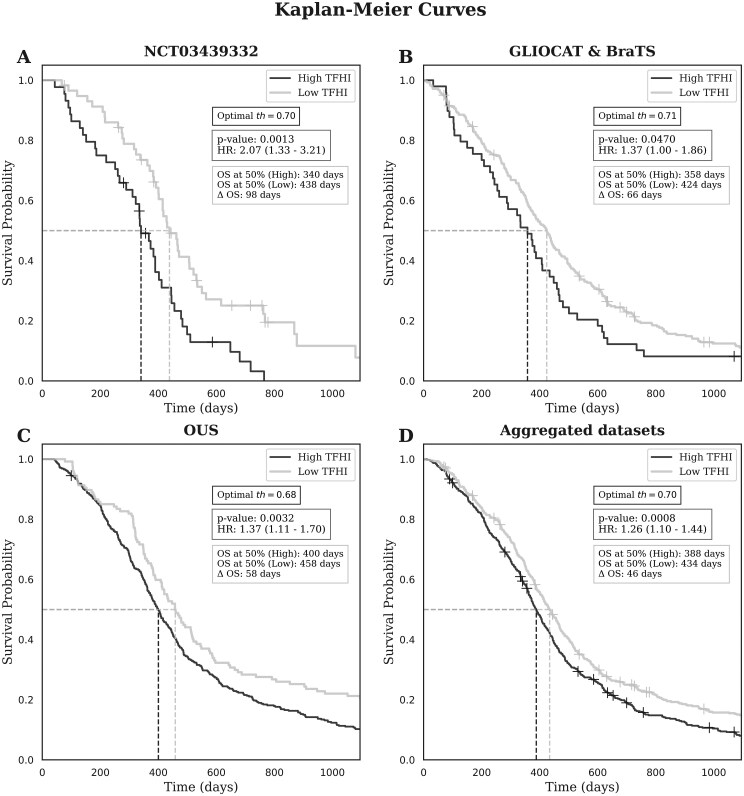
Kaplan-Meier curve for the optimal threshold obtained through the optimization of the C-index associated with Cox regression (A) NCT03439332, (B) GLIOCAT & BraTS, (C) OUS, and (D) all datasets combined, for the TFHI, which biomarker separated high and low survivors. Additionally, the median survival time (when 50% of patients have died) was calculated for each group and the difference between them (Δ OS). To improve the visual interpretation of survival differences, all curves were truncated at 3 years (1095 days).

Figure 4 provides an overview of the results from univariate analysis. Specifically, it presents the optimal thresholds for distinguishing between patients with high and low survival rates based on Cox regression, thereby establishing a link between nCET segmentation and OS. For the TFHI, the optimal threshold derived for each dataset (th = 0.70, 0.71, 0.68, 0.70) significantly differentiated the high-survival and low-survival groups (*P* < .05) with differences in median OS ranging from 46 to 98 days. At this threshold, the likelihood of mortality in the low-survival group was approximately twice as high as in the high-survival group in the NCT03439332 dataset (HR: 2.07 [95% CI: 1.33-3.21]; *P* = .0013, c-index = 0.6). Notably, validating the threshold across independent datasets reinforces its robustness. In the GLIOCAT & BraTS and OUS datasets, while the differences in HR were less pronounced, they remained significant (HR: 1.37 [95% CI: 1.00-1.86]; *P* = .047; c-index = 0.58 and HR: 1.37 [95% CI: 1.11-1.70]; *P* = .0032; c-index = 0.59, respectively). Finally, the analysis combining all datasets confirms the reliability of this partitioning, yielding an HR of 1.26 [95% CI: 1.10-1.44]; *P* = .0008; c-index = 0.6 and establishing a definitive threshold of 0.70 for significantly distinguishing high-survival and low-survival groups.

Moreover, the volumetric ratio of the enhancing tumor to edema (CET/edema) yielded significant associations with OS across all four experiments. The strongest result was observed in the NCT03439332 dataset (*P* = .0005, HR = 2.41 [1.47-3.98], ΔOS = 94), followed by the aggregated dataset (*P* = .0008, HR = 1.38 [1.14-1.68], ΔOS = 54). While the CET/edema ratio is correlated with OS—as expected given its established clinical relevance and the fact that the CET is routinely targeted during surgical resection—this variable was excluded from the final multivariate analysis, which specifically focused on the infiltrative nCET captured by the TFHI. Notably, the TFHI demonstrated a comparable prognostic performance to CET/edema, highlighting that the nCET region—although typically not completely resected—may carry equivalent prognostic value.

The Kaplan–Meier curves at the cohort-specific optimal threshold (Figure 4) show that patients with lower TFHI have longer overall survival (*P* < .05). Median OS differences—defined as the time point at which the estimated survival probability reaches 50%—were 98 days (NCT03439332), 66 days (GLIOCAT & BraTS), 58 days (OUS), and 46 days in the pooled cohort. At this optimal threshold, TFHI separates patients into two prognostic groups with significantly different survival, with a median OS gap exceeding 1.5 months.

#### Multivariate Analysis

Table 1 shows the effect of the different variables on OS based on Cox regression. The NCT03439332 and GLIOCAT datasets were used to determine if other variables already explain the TFHI prognostic relevance. Cox regression was first implemented including clinical variables, perfusion information and edema volume and it was noted that the significant variable is *MGMT* methylation status (HR: 1.92 [95% CI: 1.39-2.64]; *P* = .00006). In contrast, sex, age, perfusion and edema volume did not contribute to the model performance.

**Table 1.** Cox regression of clinical, perfusion and volumetric predictors (± TFHI) in NCT03439332 and GLIOCAT cohorts^a^

Features	Excluding TFHI		Including TFHI			
	CV + perfusion + volumetry		CV + perfusion		All variables	
	HR (95% CI)	*P*-value	HR (95% CI)	*P*-value	HR (95% CI)	*P*-value
Age	1.22 [0.62- 2.40]	.57	1.34 [0.67-2.52]	.45	1.34 [0.68-2.52]	.43
Gender	1.04 [0.77-1.40]	.82	1.09 [0.73-1.39]	.80	1.05 [0.77-1.41]	.80
*MGMT*	1.92 [1.39-2.64]	.00006**	1.91 [1.42-2.66]	.00005**	1.93 [1.40-2.66]	.00006**
rCBV_max-nCET_	2.21 [0.73-6.65]	.16	2.33 [0.80-6.62]	.10	2.50 [0.80-7.34]	.08
Edema volume	0.73 [0.39-1.37]	.33	-	-	1.24 [0.59-2.66]	.59
TFHI	-	-	2.60 [1.22-5.50]	.012*	3.02 [1.20-7.56]	.017*
C-index	0.57		0.58		0.59	

a Categorical coding and reference levels: MGMT methylated = 0 (reference); unmethylated = 1 (HR vs methylated). Gender male = 0 (reference); female = 1 (HR vs male). Continuous covariates: age, rCBV_max-nCET_, edema volume, TFHI (treated as continuous in multivariate models).

Abbreviations: CI = confidence interval; CV = clinical variables (age, gender, MGMT promoter methylation status); HR = hazard ratio; nCET = non-contrast-enhancing tumor; rCBV = relative cerebral blood volume; TFHI = T2/FLAIR Heterogeneity Index.

* *P* < .05;

** *P* < .01.

When the TFHI was added to the same multivariate framework, its influence produced a significant contribution when analyzed with clinical variables and perfusion and then including volumetric variables. The TFHI-associated hazard ratio increases when volumetric information is added, implying that the edema volume enhance—but does not replace—the prognostic content of the TFHI; it still yields a significant *P*‑value together with a larger effect size (HR: 3.02 [95% CI: 1.20-7.56]; *P* = .017). The methylation status of *MGMT* remains virtually unchanged across all experiments, suggesting that it does not represent a shared source of variation with the TFHI, edema volume, or perfusion metrics. Additionally, to verify that the information provided by the TFHI is not just related to any of its components alone, Cox regression was also performed on the volume of nCET and edema separately, showing that individually they are not significant (*P *> .05).

We further examined the partial/conditional effect of TFHI on OS using the full multivariable model weights and ­stratifying by MGMT status. Consistent with Table 1, the ­incremental effect of TFHI was essentially null in MGMT-unmethylated patients—changes in TFHI did not materially alter predicted survival relative to the baseline model. In contrast, among MGMT-methylated patients, lower TFHI values were associated with longer OS across the survival curve, indicating that TFHI contributes meaningful prognostic separation in this subgroup. These patterns align with the overall multivariable associations and support TFHI’s complementary prognostic contribution beyond standard covariates.

### Histopathological Sampling of MRI-Defined nCET

**Figure 5.**
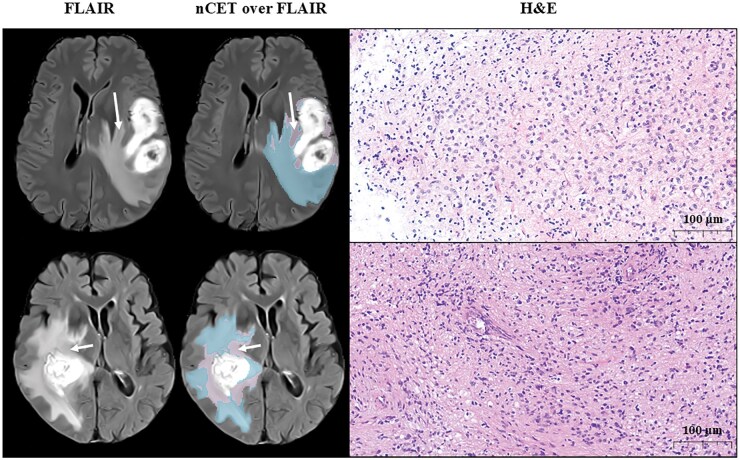
MRI–histology correspondence for nCET-targeted regions in two glioblastoma cases. For each case, we show the preoperative FLAIR MRI, the FLAIR with overlays of putative nCET (pink) and vasogenic edema (blue), and the corresponding H&E histology (scale bar: 100 µm). Arrows indicate the approximate sampling site planned with neuronavigation (Brainlab Elements or Medtronic); sampling was performed before resection. Navigation relied on preoperative MRI in all cases, with intraoperative ultrasound used when available to mitigate brain shift. The H&E sections display hypercellular proliferation with irregular, medium-sized nuclei, consistent with infiltrating glioblastoma. These examples are illustrative and do not constitute a comprehensive biological validation.

To provide biological context beyond MRI, we present in Figure 5 two glioblastoma cases in which tissue was sampled from MRI-defined nCET using navigation with preoperative nCET masks and intraoperative imaging (ultrasound when available) (SINUE, NCT07111195). In both patients, the putative nCET is located in regions that, on H&E, show hypercellularity with irregular, medium-sized nuclei, consistent with infiltrating glioblastoma. These examples support the plausibility of the MRI-defined nCET compartment; they are illustrative and do not constitute a comprehensive biological validation.

## Discussion

Our study presents a reproducible methodology for segmenting peritumoral edema into imaging-defined subregions in patients with GB, distinguishing a putative nCET–related subregion from vasogenic edema based on commonly accessible conventional MRI sequences. As an imaging-based construct, it reflects T2/FLAIR heterogeneity but does not inherently imply the presence of a discrete cellular-level boundary. The method is implemented as a standalone module on the ONCOhabitats server and is freely accessible for research use. We validated the clinical significance of the putative nCET region delineated by assessing the prognostic capabilities of a derived biomarker, TFHI, in an GB multicentric datasets.

The nCET region has been reported to harbor infiltrative tumor cells despite being outside the contrast-enhancing area that defines the tumor core.^7^^,^^8^ Accurate segmentation of this region based on T2 and FLAIR MRIs holds potential for understanding the complex architecture of GB. Our results demonstrate that 97% of patients exhibited a practical intensity difference between the imaging-defined subregion hypothesized to correspond to nCET and vasogenic edema. As qualitative biological context beyond MRI, we include two putative nCET-targeted H&E cases, sampled using navigation with preoperative nCET masks and intraoperative ultrasound (when available). Both showed hypercellularity with irregular, medium-sized nuclei consistent with GB infiltration. These examples support the plausibility of the MRI-defined nCET compartment but are illustrative and do not constitute a comprehensive biological validation.

The proposed TFHI biomarker was significantly associated with OS, offering prognostic information beyond conventional volumetric, perfusion, and genetic parameters. In particular, TFHI captures a part of the infiltrative component of nCET relative to edema based on MRI—a region that may remain after surgical resection. Remarkably, the prognostic performance of the TFHI (ie the nCET/Edema ratio) was found to be nearly equivalent to that of the enhancing tumor CET/edema ratio. This is clinically relevant, as the CET region is routinely targeted and resected, whereas nCET is generally partially left in situ. That a non-resected, imaging-defined nCET compartment demonstrates prognostic power comparable to a surgically removed region underscores the clinical relevance of nCET and highlights the TFHI’s potential to guide both outcome prediction and therapeutic decision-making.

Our findings underscore the prognostic value of the TFHI and its potential to complement existing clinical tools by quantifying T2/FLAIR signal heterogeneity within the edema—a region that often persists after resection. The consistency of the optimal threshold (th = 0.7) across independent datasets supports its robustness, and stratification at this threshold consistently distinguished groups with >1.5-month differences in median OS. While a higher TFHI is consistent with more diffuse nCET, this should not be interpreted as cellular-level proof of infiltration; further biological validation would be required to establish histologic correspondence.

*MGMT* methylation status was identified as a key predictor of OS, in line with established evidence.^31^ Importantly, among patients with *MGMT*-methylated tumors—a group generally associated with more favorable outcomes—the extent of imaging-defined nCET diffusely invading the edema, as captured by the TFHI, emerged as a particularly relevant prognostic factor. In the final multivariable models, adding TFHI to clinical ± imaging covariates produced a modest but consistent gain in discrimination, with c-index increases of ∼0.01-0.02 (eg 0.57-0.59 in Table 1). This observation suggests that a high TFHI may denote a more aggressive and infiltrative tumor phenotype, even within a biologically favorable subgroup. The capacity of the TFHI to quantify this infiltrative component adds complementary prognostic information beyond conventional markers, such as CET volume, extent of resection, *MGMT* methylation status and *IDH1* mutation status,^2^^,^^11^^,^^19^^,^^31^ supporting its potential utility for patient stratification in clinical trials and for tailoring treatment strategies. Additionally, *MGMT* methylation retained significance in the multivariate model, further reinforcing their clinical relevance.

Despite these promising results, the primary limitation of our study is the lack of gold standard for nCET segmentation. While the radiographic definition of nCET was employed, the clinical significance of the segmentation was corroborated through its association with OS and multi Cox regression analyses. Future work will address this limitation through full histopathological validation and a dedicated analysis of paired pre- and early postoperative MRI to quantify the proportion of preoperative nCET that remains as residual nCET and to evaluate its association with patient outcomes.

From a clinical perspective, the findings could have relevant implications for surgical planning. Current guidelines advocate supramaximal resection to improve survival in GB patients.^5^^,^^6^ However, there is no consensus on the extent of resection, particularly concerning the edema surrounding the tumor core, with less than 5 cm³ of nCET needing to remain for the procedure to be considered supramaximal.^6^ Our methodology provides a reproducible, imaging-based tool to map T2/FLAIR heterogeneity within the apparent edema—consistent with possible non-enhancing disease—thereby offering neurosurgeons additional preoperative information to refine strategies while balancing oncologic benefit and functional safety.

## Data Availability

The main results presented in this study can be reproduced using the publicly available BraTS dataset, which can be accessed through the Multimodal Brain Tumor Segmentation Challenge repository (https://www.med.upenn.edu/cbica/brats), together with the open-access software tools provided through the ONCOhabitats platform. The remaining datasets (OUS, Gliocat, and the NCT03439332) contain sensitive patient-level information and are owned by the respective institutions. Access to these data is restricted but may be granted upon reasonable request to the corresponding centres and subject to approval by the relevant institutional ethics committees.
